# Case report: The effect of induction targeted therapies in stage III driver mutants non-small cell lung cancer

**DOI:** 10.3389/fonc.2024.1286116

**Published:** 2024-11-13

**Authors:** Waleed Kian, Belal Krayim, Betsy Giles, Nasim A. Elkiaan, Amjad Idris, Daniel Fink, Nir Peled, Laila C. Roisman

**Affiliations:** ^1^ Helmsley Cancer Center, Shaare Zedek Medical Center, Jerusalem, Israel; ^2^ Institute of Oncology, Samson Assuta Ashdod University Hospital, Ashdod, Israel; ^3^ Ben-Gurion University of the Negev, Be’er Sheva, Israel; ^4^ Thoracic Surgery Department, Shaare Zedek Medical Center, Jerusalem, Israel

**Keywords:** brigatinib, osimertinib, neoadjuvant, *ALK*, *EGFR*, lung cancer

## Abstract

**Background:**

Over the past decade, progress in the diagnosis and treatment of Non-Small Cell Lung Cancer (NSCLC) has led to the identification of many targeted mutations. This has enhanced PFS and OS in both advanced and early-stage NSCLC. The current standard of care for stage III NSCLC varies, and it may combine chemotherapy with either immunotherapy or radiotherapy. This study evaluated the role of induction targeted therapies in patients with driver mutations and inoperable NSCLC.

**Methods:**

This is a single-center, retrospective study assessing the efficacy of targeted therapy in resectable stage III NSCLC patients who are *EGFR* or *ALK*-positive, using patient records, PET-CT, brain MRI staging, and mediastinal lymph node evaluation.

**Results:**

Between January 2020 and February 2024, we identified four patients with either *EML4-ALK* fusions (2/4) or *EGFR* mutations (2/4) who underwent treatment with brigatinib or osimertinib before surgery. All patients experienced clinical benefits. Of the two patients with *ALK* fusion, one responded almost completely, while the other exhibited a notable partial response. Among the patients with *EGFR* mutations, one had a complete response and the other displayed a significant partial response. All four patients subsequently underwent lobectomy surgical resection.

**Conclusions:**

This case series highlights the potential of targeted therapies for resectable NSCLC in the neoadjuvant setting. Further research is required to confirm their benefits, assess their safety and efficacy, and determine optimal timing and sequencing.

## Introduction

Lung cancer, the most common form of cancer globally, has the highest mortality rate among all cancers. Smoking is the primary risk factor. Lung cancer is broadly classified into two types: non-small cell lung cancer (NSCLC) and small cell lung cancer (SCLC). NSCLC, which makes up around 85% of cases, includes subtypes such as adenocarcinoma, squamous cell carcinoma, and large cell carcinoma (1). Technological advancements and immunohistochemical techniques have enabled personalized treatments based on specific driver mutations in individual tumors, providing new hope for lung cancer patients (2).

Patients who undergo surgical resection are still at a high risk of relapse. To address this concern, adjuvant and neoadjuvant chemotherapy have been studied extensively and have shown promising results in improving disease-free survival (DFS) and overall survival (OS) (3–5). In addition to chemotherapy, recent trials have explored the potential benefits of adjuvant immunotherapy and targeted therapy in patients with early-stage disease. One notable study is a phase III trial that compared adjuvant atezolizumab to the standard of care (SOC) in patients with resected stage II or III disease and PD-L1 expression of 1% or greater. The results of this trial demonstrated a significant improvement in DFS for patients with PD-L1 >1% and OS, particularly for those with high PD-L1 expression (>50%) (6, 7). Another important trial investigated the use of adjuvant pembrolizumab versus placebo in patients with stage IB-III, regardless of tumor proportion score PD-L1 expression. This study also revealed a notable enhancement in DFS (8).

Finally, the ADAURA trial, a phase III trial comparing adjuvant osimertinib to SOC, demonstrated an improvement in DFS and OS for patients with *EGFR* mutant NSCLC (9, 10). Furthermore, the Phase III ALINA trial also showed an improvement in DFS with the addition of adjuvant alectinib (11). These results, along with those from other ongoing trials, highlight the integration of immunotherapy and targeted therapies in the treatment approach for patients with surgically resected NSCLC. As a result, the FDA and EMA have granted approvals for specific populations.

In the neoadjuvant setting, a phase III trial comparing chemotherapy and nivolumab with chemotherapy alone demonstrated an improvement in the rate of pathological complete response and event-free survival in patients with stage IB-IIIA disease (12). Neoadjuvant trials have explored new endpoints, such as major and complete pathological response, which could potentially serve as surrogate endpoints in future trials. We recently published a Phase II trial focusing on neoadjuvant Osimertinib in Stage III *EGFR*-positive NSCLC, followed by definitive radiation and/or surgery. The trial showed a high response rate of 95.2% with excellent safety, as well as a nearly 50% reduction in the radiation field (13). In light of this, we present four patients who received neoadjuvant targeted therapies for potentially resectable stage III NSCLC with oncogenic driver mutations (*EGFR* or *ALK*), with the goal of determining their efficacy in this setting.

## Methods

This document pertains to a single-center, retrospective, observational study aimed at assessing the efficacy of neoadjuvant targeted therapy in patients with potentially resectable NSCLC harboring *EGFR* or *ALK*-positive mutations. Data were extracted from patient records, including PET-CT scans, brain MRI for baseline tumor staging (according to the AJCC 8th edition), and pathological evaluation of mediastinal lymph nodes. Eligible patients demonstrated normal organ function, adequate pulmonary function, and an Eastern Cooperative Oncology Group performance status score of zero. Driver mutations were confirmed through next-generation sequencing.

Among the cohort, two patients had *ALK* fusions and two had *EGFR* mutations, all of whom received targeted tyrosine kinase inhibitor (TKI) therapies. Patients with *ALK* fusion genes were treated with brigatinib at a daily dosage of 180mg, while those with *EGFR* mutations received osimertinib at a daily dosage of 80mg. It is important to note that the off-label use of treatment in these cases was conducted as part of a local scientific project.

PET-CT scans and brain MRIs were utilized to evaluate treatment efficacy. Following induction of targeted therapy, all responsive patients underwent surgery, after which pathological response was assessed.

## Case presentation

### Case 1

**Figure 1:**
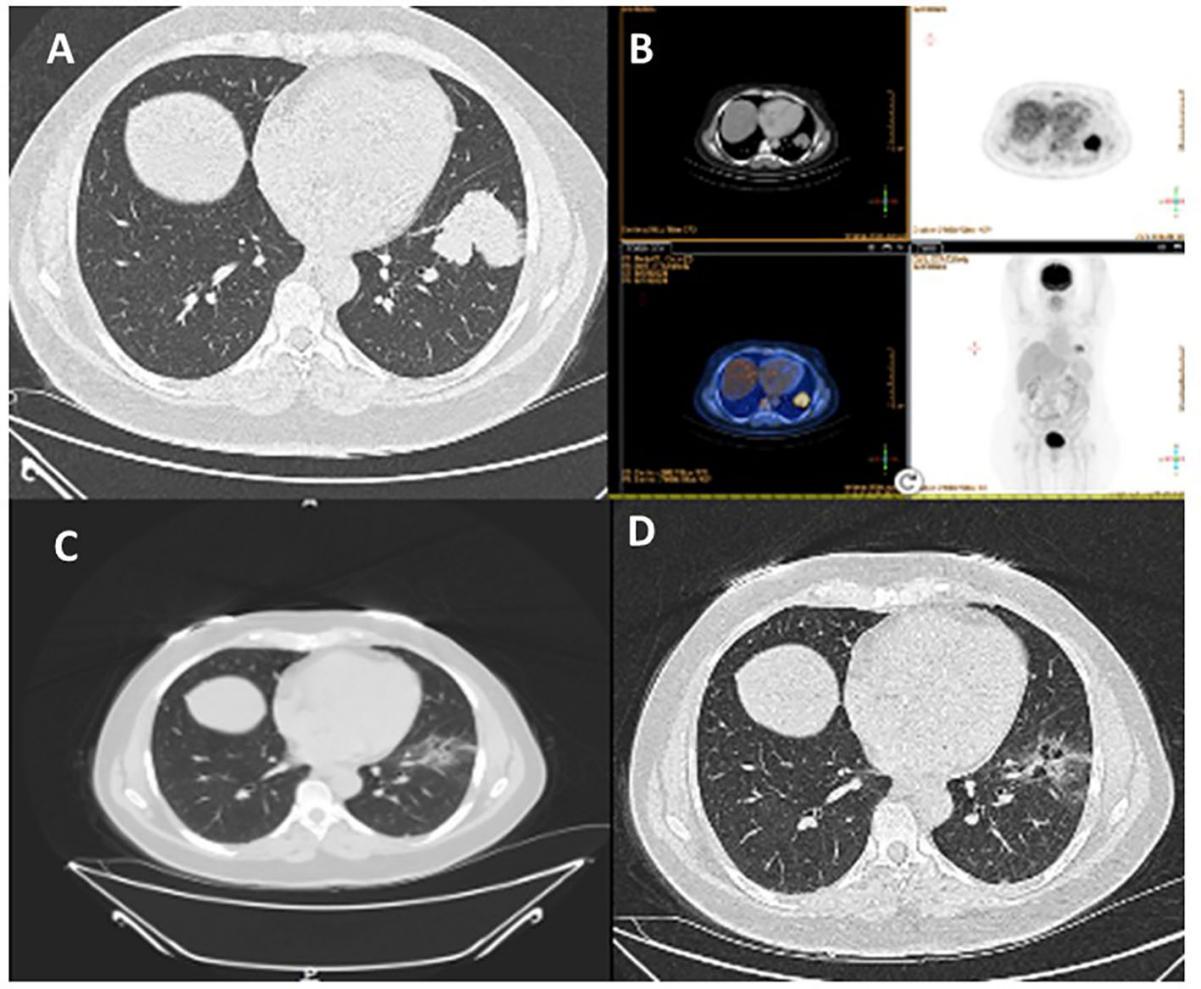
Case 1: A 51-year-old female with NSCLC-adenocarcinoma and *EML4–ALK* fusion. **(A)** Chest CT shows a 5cm mass in the left lower lobe, classified as T3N2M0. **(B)** PET-CT scan indicates no metastasis. **(C, D)** Follow-up CT chest after 6 weeks of treatment with brigatinib 180mg daily.

In August 2021, a 51-year-old non-smoking female underwent a routine imaging exam which revealed the presence of a 5 cm mass in the left lower lobe. This mass was diagnosed as adenocarcinoma of lung origin through a CT-guided biopsy. Further testing using PET-CT showed significant fluorodeoxyglucose (FDG) uptake in the left lower lobe and moderate uptake in the mediastinal lymph nodes on the same side, indicating the absence of distant metastasis (**Figure 1**). Brain MRI results were negative for intracranial metastasis.

According to the American Joint Committee on Cancer (AJCC) 8th Edition, the patient’s condition was classified as T3N2M0. To address the patient’s condition, the multidisciplinary team decided to initiate neoadjuvant treatment with brigatinib, followed by surgery. After six weeks of treatment, a chest CT showed a partial response with significant tumor shrinkage. Subsequently, the patient underwent left lower lobectomy and mediastinal lymph node dissection. The pathology report indicated a pathological response of pT1cN2 and negative Spread through air spaces (STAS). Currently, the patient is 27 months post-surgery and is undergoing adjuvant treatment with a daily dose of 90mg of brigatinib. Recent PET-CT scan and brain MRI results showed no evidence of disease, as summarized in **Table 1**.

**Table 1:** Summary of patient characteristics and treatments.

	P1	P2	P3	P4
**Age**	51	46	74	59
**Histology**	ADC	ADC	ADC	ADC
**Symptoms**	No	Cuogh	Cough/dyspnea Weight loss	No
**Smoker status**	Never	Never	Never	Never
**Stage at diagnosis**	T3N2M0	T3N2M0	T2bN2M0	T2aN2M0
**Brain mets**	NO	NO	NO	NO
**Driver mutation**	ALK-EML4 fusion	ALK-EML4 fusion	EGFR L858R&L861Q	EGFR exon 19 deletion
**PDL-1 status**	PDL-1 <1%	PDL-1 <1%	PDL-1 1-49%	PD-L1 > 50%.
**Targeted therapy**	Brigatinib 180 mg	Brigatinib 180 mg	Osimertinib 80 mg	Osimertinib 80 mg
**Duration of neoadjuvant treatment**	6 weeks	7 weeks	12 weeks	12 weeks
**Best response %**	PR	PR	PR	CR
**Surgical procedure**	VATS LLL lobectomy	VATS RLL lobectomy	VATS RUL lobectomy	VATS LUL lobectomy
**Pathlogical respnse**	T1cN2	T1cN1	pT1aN0	pCR
**Adjuvant treatment**	Brigatinib 90 mg	Brigatinib 90 mg	No	osimertinib 80mg
**DFS**	27 month	32 month	42 month	24 months
**Recurrence disease**	No	No	No	NO

ADC, Adenocarcinoma; PD-L1, Programmed death ligand; RUL, Right upper lobe; LLL, Left lower lobe; LUL, Left upper lobe; RLL, Right lower lobe; VAST, Video,assisted thoracoscopic surgery; pCR, pathological complete response; PR, partial response, CR, complete response; DFS, disease free survival.

### Case 2

**Figure 2:**
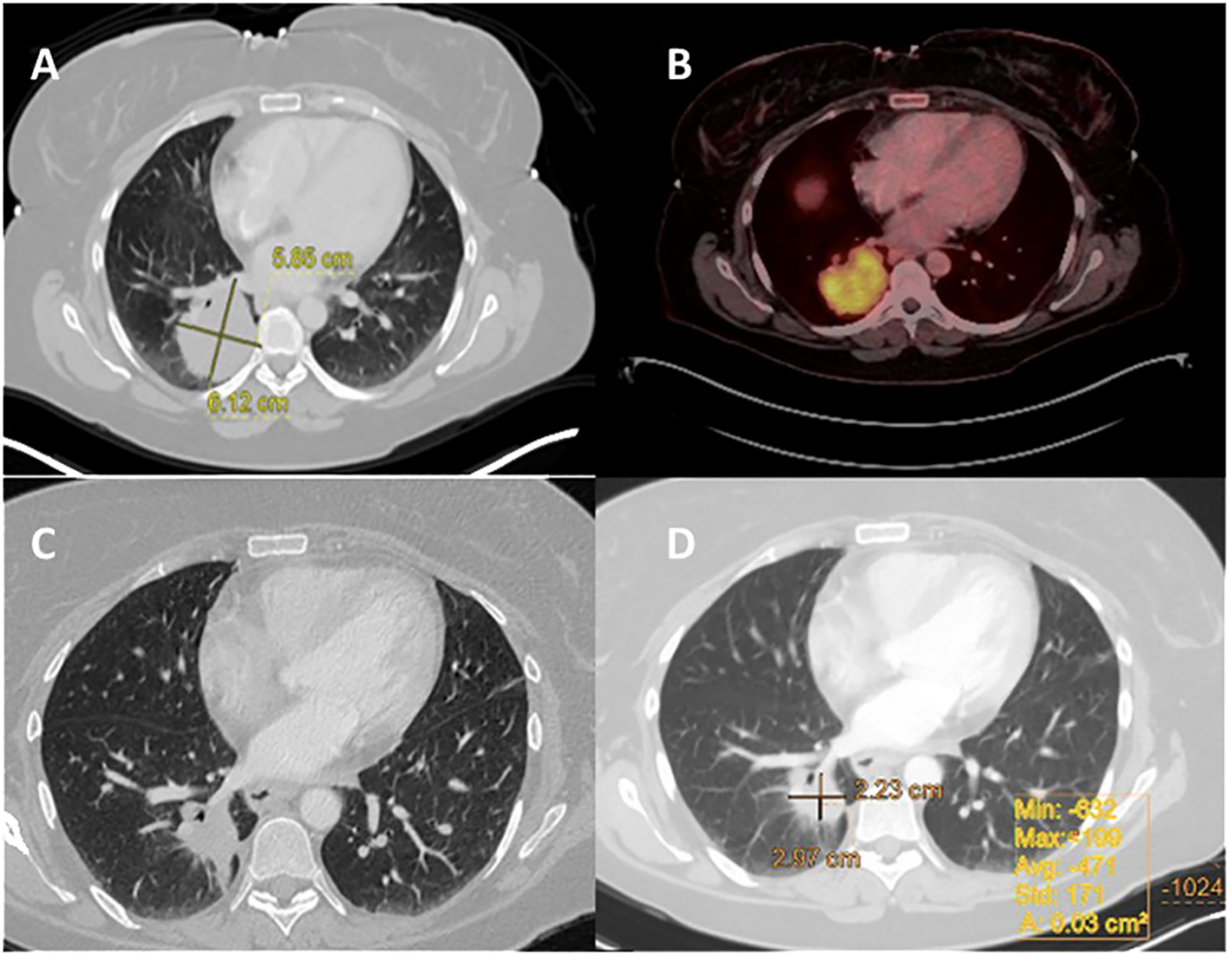
Case 2: A 46-year-old female with NSCLC-adenocarcinoma and *EML4-ALK* Fusion. **(A)** Chest CT shows a right lower lobe (RLL) mass measuring 5.5 cm, with a staging of T3N0M0. **(B)** PET CT shows FDG uptake in the RLL mass measuring 5.5 cm, without metastasis. **(C, D)** After 6 weeks of treatment with brigatinib 180 mg daily, CT chest was performed.

A 46-year-old nonsmoking female presented with a suspicious mass on a chest x-ray while hospitalized with SARS-CoV-2 in February 2021. A chest CT scan revealed a 5.5 cm mass involving the costophrenic angle in the right lower lobe. A subsequent PET-CT scan revealed high FDG uptake in the right lower lobe and moderate FDG uptake in the ipsilateral mediastinal lymph nodes but no distant metastasis (**Figure 2**). An MRI of the brain revealed no evidence of intracranial metastasis. Adenocarcinoma of the lung was confirmed by CT-guided biopsy and tissue next-generation sequencing revealed an *EML4-ALK* fusion rearrangement. The patient was classified as T3N2M0. The patient began neoadjuvant brigatinib, but experienced side effects such as fever and weakness, resulting in a 50% reduction in dosage from 180mg to 90mg, which was maintained for 7 weeks. Based on a follow-up chest CT, the patient showed a partial response to treatment, with 60% remarkable tumor shrinkage. The patient had a right lower lobectomy and mediastinal lymph node dissection (pT1cN1 pathological response, STAS negative). Following surgery, the patient received adjuvant brigatinib 90 mg once daily for 32 months, with no evidence of disease detected on PET-CT. An MRI also revealed no brain metastases, as summarized in **Table 1**.

### Case 3

**Figure 3:**
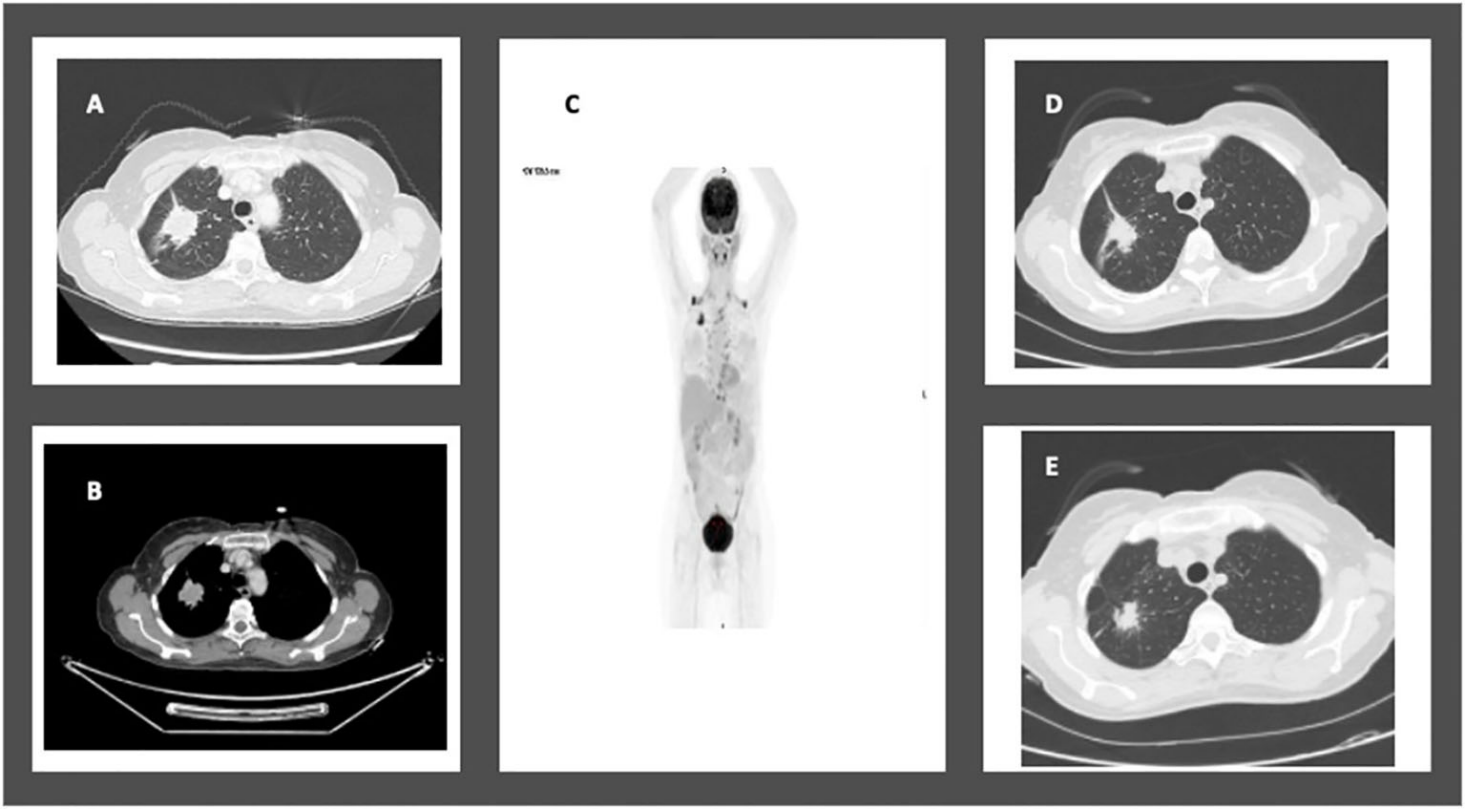
Case 4: A 74-year-old female with NSCLC-adenocarcinoma and *EGFR* exon 21 L858R mutation. **(A, B)** Chest CT showing a 3.5cm mass in the right upper lobe. **(C)** RUL mass and moderate FDG uptake in the ipsilateral mediastinal lymph nodes. **(D, E)** CT chest after 12 weeks of treatment with Osimertinib 80mg daily showing partial response.

In January 2020, a 74-year-old female non-smoker was diagnosed with a 3.5 cm mass in the right upper lobe during a routine imaging examination. A PET-CT scan revealed high FDG uptake in the right upper lobe and moderate FDG uptake in the ipsilateral mediastinal lymph nodes, without distant metastasis (**Figure 3**). Brain MRI showed no intracranial metastasis. A CT-guided biopsy revealed lung adenocarcinoma. Tissue next-generation sequencing showed an EGFR L858R and L861Q mutations. According to the AJCC 8th Edition guidelines, the patient was staged as T2bN2M0. The patient was treated with osimertinib for 12 weeks, demonstrating a partial response to treatment of 80% on chest CT. In August 2020, the patient underwent right upper lobectomy and mediastinal lymph node dissection (pT1aN0 pathological response, STAS negative). Following recovery from surgery, no adjuvant therapy was taken. After 42 months of follow-up, there was no evidence of disease on PET-CT or brain MRI, as summarized in **Table 1**.

### Case 4

**Figure 4:**
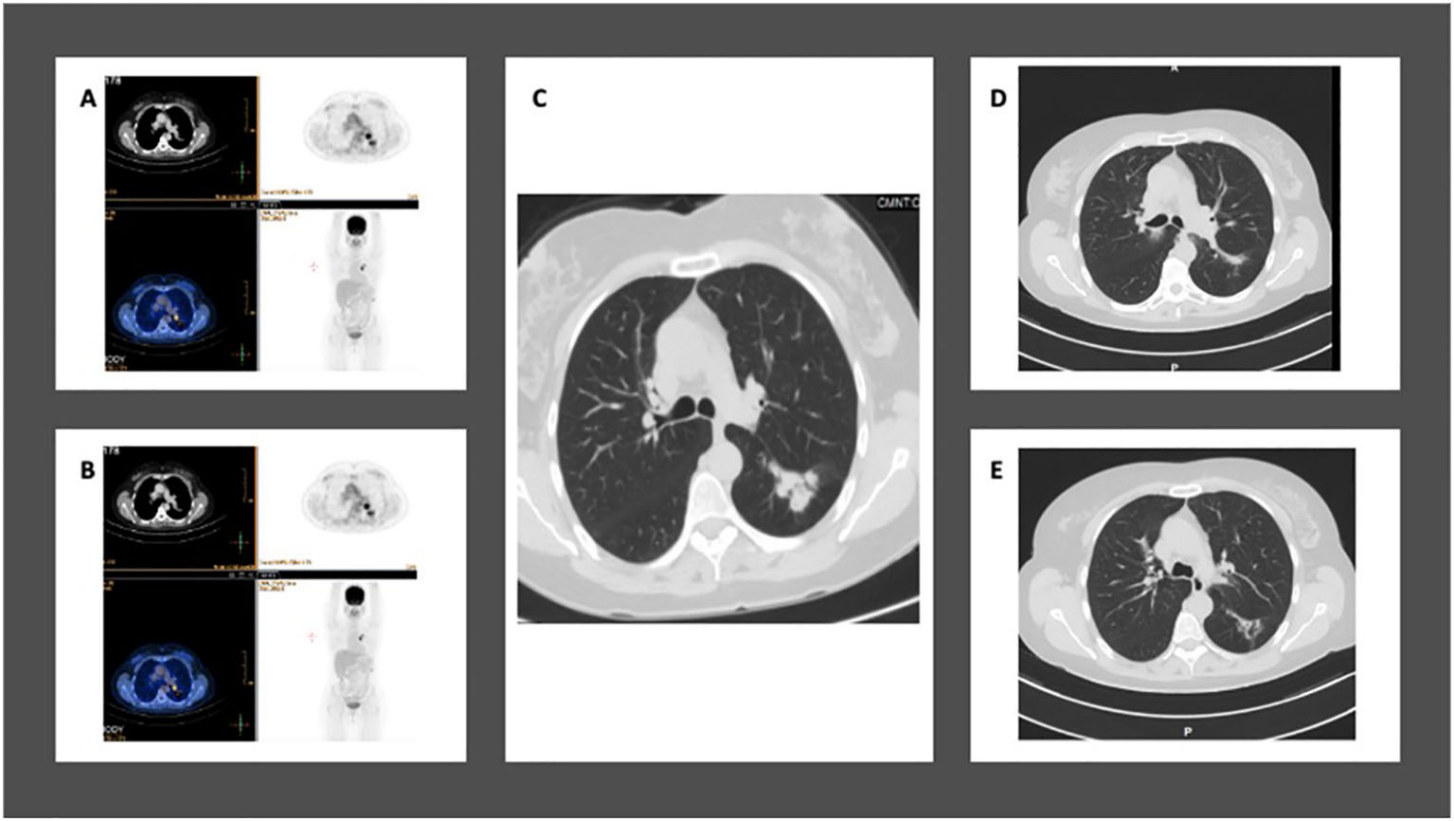
Case 5: A 59-year-old female with NSCLC-adenocarcinoma and *EGFR* exon 19 deletion. **(A, B)** PET-CT shows a 3.5 cm mass in the left lower lobe with mediastinal lymph nodes, but without distant metastasis. The staging is T2aN2M0. **(C)** Chest CT shows a mass in the left lower lobe measuring 3.5 cm. **(D, E)** After 12 weeks of treatment with Osimertinib at a daily dose of 80 mg, CT chest shows complete response.

A 59-year-old former smoker was diagnosed with a 3.7 cm mass in her left lower lung lobe during a routine imaging exam in September 2021. A PET-CT scan revealed high FDG uptake in the left upper lobe and moderate FDG uptake in both the ipsilateral and contralateral mediastinal lymph nodes, with no distant metastasis (**Figure 4**). No intracranial metastasis was detected on a brain MRI.

A CT-guided biopsy confirmed the mass to be an adenocarcinoma of lung origin. Tissue next generation sequencing revealed an *EGFR* exon 19 deletion. The patient was classified as T2aN2M0.

The patient started treatment with Osimertinib, taking an 80 mg dose daily for 12 weeks. This resulted in a radiological complete response on the PET-CT. In February 2022, she underwent a resection of the left upper lobe, achieving a pathological complete response. She continued with adjuvant Osimertinib treatment. After 24 months of follow-up, there is no sign of metastasis on her PET-CT and brain MRI, as summarized in **Table 1**.

## Results

Between January 2020 and February 2024, four enrolled participants received targeted therapy. All patients had been diagnosed with adenocarcinoma, with two Stage IIIA patients and two Stage IIIB patients. The participants characteristics shown in **Tables 1**, **2**. Representative radiologic and pathological responses after 6 to 12 weeks of brigatinib or osimertinib are shown in **Table 3**.

**Table 2:** Demographics.

Patient Characteristics (n = 4)	
Age, years	
** Median (range)**	59 (46-74)
Gender, n (%)	
** Male**	0 (0)
** Female**	4 (100)
Smoking history n (%)	
** Never smoker**	4 (100)
** Former smoker**	0 (0)
Performance status, n (%)	
** 0**	4 (100)
** 1**	0
Tumor histology, n (%)	
** Adenocarcinoma**	4 (100)
Driver -mutation Type, n (%)	
** Exon 19 deletion**	1 (25)
** Exon 21 L858R & L861Q**	1 (25)
** ALK-EML4 fusion**	2 (50)
Stage, n (%)	
** IIIA**	2 (50)
** IIIB**	2 (50)

EGFR, epidermal growth factor receptor.

**Table 3:** Radiological and pathological outcomes of induction targeted therapy.

Outcome	Osimeratinib/Brigatinib (N=4)
**Radiologic outcome; ORR (95% CI)**	100% (4)
** Complete response**	25% (1)
** Partial response**	75% (3)
** Stable disease**	0%
** Progression of disease**	0%
**Range of neoadjuvant DoT, months (95% CI)**	6-12 weeks
Pathological outcome	
** Complete pathological response**	25% (1)
** Major partial response**	25% (1)
** Partial response**	50% (2)
**Median DFS, months**	18 months
**Disease relapse**	0/4

ORR, objective response rate; DoT, duration of treatment; DFS, disease free survival.

Among the two patients who had an *ALK* fusion, one showed a radiological response of 90%, while the other showed a partial response of 60%. The first *EGFR* patient had a partial radiological response rate of about 80%, while the second patient had a complete radiological response. During neoadjuvant therapy, only one patient experienced grade 3 side effects (fever and weakness) that necessitated a dose reduction, as summarized in **Table 4**.

**Table 4:** Adverse event related to the targeted therapies osimertinib and brigatinib.

	Any grade	Grade1	Grade2	Grade3	Grade4
N=4 (%)					
Rash or acne	1 (25)	0	1 (25)	0	0
Diarrehea	3 (75)	2 (50)	1 (25)	0	0
Nausea	1 (25)	1 (25)	0	0	0
Fatigue	4 (100)	2 (50)	1 (25)	1 (25)	0
Anemia	2 (50)	0	2 (50)	0	0
Pyrexia	1(25)	0	0	1(25)	0

All the patients underwent lobectomy resection. After surgery, one patient had a major pathological response (MPR), another patient had a complete pathological response, and the other two had a partial pathological response. The patients underwent postoperative follow-up using PET-CT and brain MRI every four months. All patients showed no evidence of disease. The treatment regimen was tolerable, and no new adverse events related to the targeted therapies osimertinib and brigatinib were reported, shown in **Table 4**.

## Discussion

The efficacy of the respective targeted therapies has been confirmed for patients with metastatic NSCLC (14, 15). These confirmatory trials suggest that these treatments prolong the progression free survival and overall survival compared to chemotherapy alone or the combination of chemo-immunotherapy (16, 17). The emergence of next-generation TKIs has ignited significant interest among researchers, with encouraging signs of sustained enhancements in disease-free survival rates observed across various intervals, as demonstrated in trials such as ADAURA with osimertinib. Furthermore, these advancements have led to improved overall survival outcomes in the adjuvant treatment of EGFR-positive NSCLC (9, 10). Notably, the recent ALINA trial revealed that adjuvant alectinib, a second-generation ALK-TKI, significantly enhanced disease-free survival compared to platinum-based chemotherapy among patients with resected ALK-positive NSCLC of stage IB, II, or IIIA (18). The use of neoadjuvant targeted therapy in NSCLC remains an important topic for study as there are many advantages of administering molecular treatment with targeting molecules before planned definitive surgery to patients with non-metastatic disease (3, 12).

The exploration of neoadjuvant targeted therapy in NSCLC represents a pivotal area of investigation, offering several advantages, especially for patients with non-metastatic disease. Early-stage NSCLC management has seen notable progress, with studies indicating that neoadjuvant chemotherapy presents a viable alternative to adjuvant chemotherapy, leading to a substantial reduction in the relative risk of death, along with significant improvements in overall survival and time-to-distant recurrence (19, 20). Specifically, for stage IIIA (N2) NSCLC, several randomized-controlled trials and meta-analyses have shown a significant survival advantage with neoadjuvant chemotherapy. Preoperative chemoradiotherapy increases the proportion of complete resections (75% vs 60%), while also increasing the rate of mediastinal downstaging (46% vs. 29%, P=0.02) and pathological responses (60% vs. 20%, P=0.0001) (21). However, both treatment strategies appear to be effective.

Recent studies have reported encouraging outcomes of neoadjuvant chemo-immunotherapy in early-stage NSCLC, surpassing previous benchmarks set by neoadjuvant chemotherapy or chemoradiation alone (22). However, the role of immunotherapy in patients with oncogenic drivers remains under scrutiny, particularly due to observed low response rates in advanced disease (23, 24).

Approximately 15% of NSCLC cases present with locoregional N2 disease (stage IIIA). The optimal treatment strategies for patients with N2 disease, as well as the criteria for defining resectability, remain subjects of ongoing debate in thoracic oncology (25). While there is still controversy surrounding the definition of resectability, the management of patients with ‘unresectable’ N2 disease is more clear-cut. The current standard of care involves concurrent chemo-radiotherapy followed by maintenance therapy with durvalumab if there is no evidence of disease progression post-induction treatment, as demonstrated in the PACIFIC trial (21, 26). For patients with potentially resectable stage IIIA (N2) disease, various trimodal approaches, including surgery, perioperative chemotherapy, and radiotherapy, are being explored by multidisciplinary thoracic teams, particularly in cases where a microscopically margin-negative resection is anticipated. Significantly, several recently published phase III trials have assessed the efficacy of perioperative chemo-immunotherapy, encompassing resectable N2 diseases, demonstrating promising improvements in event-free survival and pathological complete response (27–29). Notably, the KEYNOTE 671 trial exhibited enhancements in overall survival (27). However, it is crucial to acknowledge that these trials included a limited number of patients with EGFR or ALK fusion mutations, rendering it challenging to draw definitive conclusions based on these findings.

The principal advantage of targeted therapy lies in its ability to commence treatment promptly, facilitating the reduction of micro-metastatic disease burden and potentially rendering tumors more amenable to surgery, particularly in cases of lymph node involvement or unresectable disease (30). Our case series underscores the effectiveness and reliability of targeted therapy as a perioperative treatment option for stage III NSCLC patients harboring EGFR mutations or ALK fusion. Promisingly, our findings revealed a median objective response rate of 100%, with no disease progression observed during the presurgical interval and no significant adverse events reported. Larger-scale studies are warranted to validate these findings across a broader patient population.

## Conclusion

This case series provides insights into the potential benefits of targeted therapies for locally advanced non-small cell lung cancer (NSCLC) in the neoadjuvant setting. The findings suggest that the use of targeted therapies in this context could be a promising approach to improve treatment outcomes for NSCLC patients.

However, while these results are certainly encouraging, more research is necessary to fully establish the role of targeted therapies in the neoadjuvant setting for NSCLC. For example, further studies are needed to verify the effectiveness and safety of these treatments, and to develop a better understanding of the optimal timing and sequencing of such therapies.

Overall, the findings of this case series underscore the importance of ongoing research into new and innovative therapeutic approaches for NSCLC and suggest that targeted therapies may have a key role to play in improving outcomes for patients with this challenging disease.

## Limitations

This case study has some limitations that should be taken into consideration. Firstly, the number of patients included in the study is relatively small, which may limit the generalizability of the results. Secondly, the follow-up period for these patients is relatively short. Thirdly, it is important to acknowledge that in actual clinical settings, postoperative patients who receive adjuvant treatments cannot be controlled compulsorily.

## Data Availability

The raw data supporting the conclusions of this article will be made available by the authors, without undue reservation.
